# Supplementary material for: Mammal responses to human recreation depend on landscape context
Source: PLoS One. 2024 Jul 18;19(7):e0300870. doi: 10.1371/journal.pone.0300870 (PMC11257333; doi:10.1371/journal.pone.0300870)
Supplement: S1 Table — Photo were obtained using the ABMI standard operating procedures protocol (Alberta Biodiversity Monitoring Institute. 2016). Also shown are the HMSC model diagnostics. Only species with a total of at least 60 independent detections (grey shaded) were included in the analysis, as there were problems with HMSC model convergence for species with fewer detections. The effective sampling size (Eff n) is the average effective sample size across all parameters for each species for each model chain. The Gelman and Rubin’s Potential Scale Reduction Factor (PSRF) is measured across all parameters estimated for each species and assesses the distribution of samples from model chains; as a rule of thumb, the mean should be <1.1 to indicate model convergence. (DOCX) [file pone.0300870.s001.docx]

***S1 Table:*** *Summary of independent (30-minute threshold) camera trap detections (Dets) and proportion of camera trap sites with at least 1 detection (Prop) for each species in the Castle (n = 64) and Bighorn (n = 91) sampling areas. Photo were obtained using the ABMI standard operating procedures protocol (Alberta Biodiversity Monitoring Institute. 2016). Also shown are the HMSC model diagnostics. Only species with a total of at least 60 independent detections (grey shaded) were included in the analysis, as there were problems with HMSC model convergence for species with fewer detections. The effective sampling size (Eff n) is the average effective sample size across all parameters for each species for each model chain. The Gelman and Rubin's Potential Scale Reduction Factor (PSRF) is measured across all parameters estimated for each species and assesses the distribution of samples from model chains; as a rule of thumb, the mean should be <1.1 to indicate model convergence.*

|  |  | Castle |  | Bighorn |  |  |  |  |
| --- | --- | --- | --- | --- | --- | --- | --- | --- |
| Species | **Common name** | **Dets** | **Prop** | **Dets** | **Prop** | **Eff n** | **PSRF.Mean** | **PSRF.Max** |
| *Odocoileus virginianus* | White-tailed Deer | 2296 | 0.92 | 3503 | 0.96 | 240 | 1 | 1.02 |
| *Lepus americanus* | Snowshoe Hare | 2180 | 0.69 | 975 | 0.52 | 220 | 1.01 | 1.04 |
| *Odocoileus hemionus* | Mule Deer | 735 | 0.90 | 533 | 0.62 | 243 | 1.01 | 1.02 |
| *Alces alces* | Moose | 657 | 0.93 | 201 | 0.52 | 233 | 1.01 | 1.02 |
| *Ursus americanus* | Black Bear | 611 | 0.95 | 425 | 0.73 | 241 | 1 | 1.02 |
| *Vulpes vulpes* | Red Fox | 504 | 0.67 | 456 | 0.63 | 213 | 1.01 | 1.03 |
| *Tamiasciurus hudsonicus* | Red Squirrel | 450 | 0.46 | 413 | 0.57 | 212 | 1.01 | 1.08 |
| *Canis latrans* | Coyote | 293 | 0.66 | 345 | 0.57 | 229 | 1.01 | 1.04 |
| *Cervus canadensis* | Elk | 257 | 0.62 | 38 | 0.13 | 203 | 1.01 | 1.06 |
| *Ursus arctos* | Grizzly Bear | 211 | 0.85 | 144 | 0.51 | 226 | 1.01 | 1.02 |
| *Spermophilus columbianus* | Columbian Ground Squirrel | 165 | 0.21 | 0 | 0.00 | 150 | 1.03 | 1.1 |
| *Martes americana* | Marten | 87 | 0.52 | 185 | 0.48 | 192 | 1.04 | 1.43 |
| *Lynx canadensis* | Canada Lynx | 86 | 0.39 | 187 | 0.48 | 151 | 1.04 | 1.26 |
| *Canis lupus* | Gray Wolf | 66 | 0.44 | 165 | 0.57 | 211 | 1.01 | 1.02 |
| *Puma concolor* | Cougar | 39 | 0.31 | 87 | 0.29 | 209 | 1.01 | 1.05 |
| *Ovis canadensis* | Bighorn Sheep | 51 | 0.07 | 8 | 0.07 |  |  |  |
| *Mephitis mephitis* | Striped Skunk | 40 | 0.25 | 9 | 0.08 |  |  |  |
| *Odocoileus spp* | Unidentified Deer | 39 | 0.34 | 148 | 0.64 |  |  |  |
| *Lynx rufus* | Bobcat | 36 | 0.18 | 1 | 0.01 |  |  |  |
| *Gulo gulo* | Wolverine | 25 | 0.15 | 16 | 0.08 |  |  |  |
| *Taxidea taxus* | American Badger | 18 | 0.08 | 0 | 0.00 |  |  |  |
| *Martes pennanti* | Fisher | 9 | 0.13 | 9 | 0.07 |  |  |  |
| *Spermophilus richardsonii* | Richardson's Ground squirrel | 7 | 0.02 | 0 | 0.00 |  |  |  |
| *Ursus spp* | Unidentified bear | 6 | 0.10 | 4 | 0.04 |  |  |  |
| *Glaucomys sabrinus* | Northern Flying Squirrel | 5 | 0.03 | 1 | 0.01 |  |  |  |
| *Mustela erminea* | Ermine | 5 | 0.05 | 0 | 0.00 |  |  |  |
| *Rodentia spp* | Unidentified Rodent | 5 | 0.05 | 0 | 0.00 |  |  |  |
| *Castor canadensis* | American Beaver | 5 | 0.02 | 0 | 0.00 |  |  |  |
| *Mustela frenata* | Long-tailed Weasel | 4 | 0.05 | 0 | 0.00 |  |  |  |
| *Erethizon dorsatum* | North American porcupine | 3 | 0.03 | 0 | 0.00 |  |  |  |
| *Leporidae spp* | Unidentified Hare | 1 | 0.02 | 20 | 0.10 |  |  |  |
| *Procyon lotor* | Raccoon | 2 | 0.03 | 0 | 0.00 |  |  |  |
| *Sciuridae spp* | Unidentified Squirrel | 2 | 0.03 | 1 | 0.01 |  |  |  |
| *Canis spp* | Unidentified Canid | 1 | 0.02 | 34 | 0.22 |  |  |  |
| *Marmota monax* | Groundhog | 1 | 0.02 | 2 | 0.02 |  |  |  |
| *Oreamnos americanus* | Mountain Goat | 1 | 0.02 | 0 | 0.00 |  |  |  |
| *Tamias minimus* | Least Chipmunk | 1 | 0.02 | 0 | 0.00 |  |  |  |
| *Marmota caligata* | Hoary Marmot | 0 | 0.00 | 3 | 0.03 |  |  |  |
| *Muridae spp* | Unidentified Mouse | 0 | 0.00 | 2 | 0.01 |  |  |  |
| *Mustela spp* | Unidentified Weasel | 0 | 0.00 | 23 | 0.16 |  |  |  |
| *Neovison vison* | American Mink | 0 | 0.00 | 1 | 0.01 |  |  |  |
| *Equus caballus* | Horse | 0 | 0.00 | 11 | 0.07 |  |  |  |
