# Supplementary material for: Mammal responses to human recreation depend on landscape context
Source: PLoS One. 2024 Jul 18;19(7):e0300870. doi: 10.1371/journal.pone.0300870 (PMC11257333; doi:10.1371/journal.pone.0300870)
Supplement: S2 Table — (DOCX) [file pone.0300870.s002.docx]

**S2 Table:** *95% Bayesian credible intervals from the HMSC model.*

| Variable | Species | 5% | 95% | Mean |
| --- | --- | --- | --- | --- |
| (Intercept) | Black Bear | **-4.15459** | **-1.79** | **-2.96** |
| (Intercept) | Canada Lynx | **-3.13** | -0.75 | -1.94 |
| (Intercept) | Cougar | **-5.52** | -2.08 | -3.70 |
| (Intercept) | Coyote | **-3.32** | -1.06 | -2.16 |
| (Intercept) | Elk wapiti | **-8.18** | -3.94 | -5.86 |
| (Intercept) | Gray Wolf | **-3.91** | -1.46 | -2.62 |
| (Intercept) | Grizzly bear | **-3.89** | -1.59 | -2.72 |
| (Intercept) | Ground squirrel | **-16.54** | -6.56 | -11.18 |
| (Intercept) | Marten | **-2.26** | 0.22 | -1.02 |
| (Intercept) | Moose | **-3.47** | -1.40 | -2.41 |
| (Intercept) | Mule deer | **-2.86** | -0.14 | -1.48 |
| (Intercept) | Red fox | **-3.12** | -0.81 | -1.95 |
| (Intercept) | Red Squirrel | **-4.21** | -1.45 | -2.80 |
| (Intercept) | Snowshoe Hare | **-4.00** | -1.00 | -2.50 |
| (Intercept) | White-tailed deer | **-0.59** | 1.40 | 0.38 |
| Strava | Black Bear | **-0.02** | 0.03 | 0.00 |
| Strava | Canada Lynx | **-0.03** | 0.02 | -0.01 |
| Strava | Cougar | **-0.05** | 0.01 | -0.02 |
| Strava | Coyote | **-0.03** | 0.02 | -0.01 |
| Strava | Elk wapiti | **-0.05** | 0.03 | -0.01 |
| Strava | Gray Wolf | **-0.04** | 0.01 | -0.01 |
| Strava | Grizzly bear | **-0.04** | 0.01 | -0.01 |
| Strava | Ground squirrel | **-0.11** | 0.05 | -0.03 |
| Strava | Marten | **-0.03** | 0.03 | 0.00 |
| Strava | Moose | **-0.04** | 0.01 | -0.02 |
| Strava | Mule deer | **-0.04** | 0.02 | -0.01 |
| Strava | Red fox | **-0.03** | 0.02 | 0.00 |
| Strava | Red Squirrel | **-0.04** | 0.01 | -0.01 |
| Strava | Snowshoe Hare | **-0.05** | 0.01 | -0.02 |
| Strava | White-tailed deer | **-0.03** | 0.03 | 0.00 |
| Distance to trail | Black Bear | **-0.15** | 0.55 | 0.21 |
| Distance to trail | Canada Lynx | **-0.12** | 0.55 | 0.22 |
| Distance to trail | Cougar | **-0.77** | 0.16 | -0.30 |
| Distance to trail | Coyote | **-0.41** | 0.25 | -0.08 |
| Distance to trail | Elk wapiti | **-0.78** | 0.41 | -0.16 |
| Distance to trail | Gray Wolf | **-0.38** | 0.27 | -0.05 |
| Distance to trail | Grizzly bear | **-0.50** | 0.22 | -0.13 |
| Distance to trail | Ground squirrel | **-1.74** | 0.68 | -0.51 |
| Distance to trail | Marten | **-0.43** | 0.30 | -0.05 |
| Distance to trail | Moose | **-0.11** | 0.56 | 0.23 |
| Distance to trail | Mule deer | **-0.71** | 0.25 | -0.22 |
| Distance to trail | Red fox | **-0.33** | 0.34 | 0.01 |
| Distance to trail | Red Squirrel | **-0.28** | 0.49 | 0.10 |
| Distance to trail | Snowshoe Hare | **-0.34** | 0.49 | 0.08 |
| Distance to trail | White-tailed deer | **-0.11** | 0.59 | 0.25 |
| Density trail | Black Bear | **-0.11** | 1.36 | 0.65 |
| Density trail | Canada Lynx | **-0.72** | 0.81 | 0.06 |
| Density trail | Cougar | **-0.79** | 0.91 | 0.06 |
| Density trail | Coyote | **-0.35** | 1.11 | 0.39 |
| Density trail | Elk wapiti | **-0.99** | 0.94 | -0.01 |
| Density trail | Gray Wolf | **-0.37** | 1.07 | 0.36 |
| Density trail | Grizzly bear | **-0.37** | 1.07 | 0.35 |
| Density trail | Ground squirrel | **-2.24** | 1.30 | -0.39 |
| Density trail | Marten | **-0.39** | 1.12 | 0.38 |
| Density trail | Moose | **-0.31** | 1.11 | 0.40 |
| Density trail | Mule deer | **-0.21** | 1.41 | 0.60 |
| Density trail | Red fox | **-0.13** | 1.32 | 0.60 |
| Density trail | Red Squirrel | **-0.47** | 1.08 | 0.31 |
| Density trail | Snowshoe Hare | **-0.61** | 0.98 | 0.19 |
| Density trail | White-tailed deer | **-0.14** | 1.55 | 0.70 |
| Non-motorized trail | Black Bear | **-2.23** | 0.28 | -0.97 |
| Non-motorized trail | Canada Lynx | **-2.05** | 0.50 | -0.80 |
| Non-motorized trail | Cougar | **-2.37** | 0.27 | -1.04 |
| Non-motorized trail | Coyote | **-2.04** | 0.41 | -0.82 |
| Non-motorized trail | Elk wapiti | **-2.83** | 0.03 | -1.41 |
| Non-motorized trail | Gray Wolf | **-2.08** | 0.39 | -0.84 |
| Non-motorized trail | Grizzly bear | **-2.16** | 0.31 | -0.92 |
| Non-motorized trail | Ground squirrel | **-4.42** | -0.05 | -2.19 |
| Non-motorized trail | Marten | **-2.08** | 0.43 | -0.85 |
| Non-motorized trail | Moose | **-2.11** | 0.39 | -0.85 |
| Non-motorized trail | Mule deer | **-2.41** | 0.24 | -1.09 |
| Non-motorized trail | Red fox | **-1.87** | 0.60 | -0.65 |
| Non-motorized trail | Red Squirrel | **-2.05** | 0.48 | -0.79 |
| Non-motorized trail | Snowshoe Hare | **-1.94** | 0.59 | -0.69 |
| Non-motorized trail | White-tailed deer | **-1.90** | 0.83 | -0.52 |
| Motorized trail | Black Bear | **-0.19** | 1.93 | 0.87 |
| Motorized trail | Canada Lynx | **-0.51** | 1.51 | 0.51 |
| Motorized trail | Cougar | **-0.81** | 1.47 | 0.34 |
| Motorized trail | Coyote | **-0.21** | 1.78 | 0.79 |
| Motorized trail | Elk wapiti | **-0.87** | 1.66 | 0.41 |
| Motorized trail | Gray Wolf | **-0.44** | 1.57 | 0.57 |
| Motorized trail | Grizzly bear | **-0.22** | 1.75 | 0.77 |
| Motorized trail | Ground squirrel | **-2.49** | 1.77 | -0.28 |
| Motorized trail | Marten | **-0.50** | 1.55 | 0.54 |
| Motorized trail | Moose | **-0.17** | 1.89 | 0.86 |
| Motorized trail | Mule deer | **-0.30** | 1.91 | 0.78 |
| Motorized trail | Red fox | **-0.24** | 1.78 | 0.79 |
| Motorized trail | Red Squirrel | **-0.38** | 1.71 | 0.66 |
| Motorized trail | Snowshoe Hare | **-0.35** | 1.76 | 0.71 |
| Motorized trail | White-tailed deer | **0.12** | 2.41 | 1.27 |
| Forest % | Black Bear | **-0.19** | 0.34 | 0.07 |
| Forest % | Canada Lynx | **-0.32** | 0.28 | -0.02 |
| Forest % | Cougar | **-0.36** | 0.34 | -0.01 |
| Forest % | Coyote | **-0.70** | -0.18 | -0.44 |
| Forest % | Elk wapiti | **-0.47** | 0.37 | -0.06 |
| Forest % | Gray Wolf | **-0.17** | 0.34 | 0.08 |
| Forest % | Grizzly bear | **-0.42** | 0.09 | -0.16 |
| Forest % | Ground squirrel | **-1.04** | 0.52 | -0.26 |
| Forest % | Marten | **0.00** | 0.60 | 0.30 |
| Forest % | Moose | **-0.15** | 0.35 | 0.10 |
| Forest % | Mule deer | **-0.12** | 0.55 | 0.21 |
| Forest % | Red fox | **-0.28** | 0.27 | 0.00 |
| Forest % | Red Squirrel | **-0.19** | 0.43 | 0.12 |
| Forest % | Snowshoe Hare | **-0.74** | -0.03 | -0.38 |
| Forest % | White-tailed deer | **-0.21** | 0.28 | 0.02 |
| Type of management | Black Bear | **0.18** | 0.62 | 0.40 |
| Type of management | Canada Lynx | **-0.45** | 0.09 | -0.17 |
| Type of management | Cougar | **-0.47** | 0.11 | -0.18 |
| Type of management | Coyote | **-0.30** | 0.14 | -0.09 |
| Type of management | Elk wapiti | **0.30** | 1.00 | 0.64 |
| Type of management | Gray Wolf | **-0.54** | -0.12 | -0.33 |
| Type of management | Grizzly bear | **0.02** | 0.44 | 0.23 |
| Type of management | Ground squirrel | **0.50** | 2.08 | 1.25 |
| Type of management | Marten | **-0.56** | -0.06 | -0.31 |
| Type of management | Moose | **0.35** | 0.75 | 0.55 |
| Type of management | Mule deer | **-0.03** | 0.47 | 0.21 |
| Type of management | Red fox | **-0.35** | 0.12 | -0.10 |
| Type of management | Red Squirrel | **-0.48** | 0.06 | -0.21 |
| Type of management | Snowshoe Hare | **-0.25** | 0.41 | 0.08 |
| Type of management | White-tailed deer | **-0.42** | -0.03 | -0.23 |
| HFI | Black Bear | **-0.21** | 0.26 | 0.03 |
| HFI | Canada Lynx | **-0.18** | 0.32 | 0.07 |
| HFI | Cougar | **-0.42** | 0.21 | -0.10 |
| HFI | Coyote | **-0.09** | 0.34 | 0.13 |
| HFI | Elk wapiti | **-0.26** | 0.48 | 0.11 |
| HFI | Gray Wolf | **-0.34** | 0.14 | -0.09 |
| HFI | Grizzly bear | **-0.39** | 0.09 | -0.15 |
| HFI | Ground squirrel | **-0.83** | 0.54 | -0.14 |
| HFI | Marten | **-0.27** | 0.25 | -0.01 |
| HFI | Moose | **-0.22** | 0.24 | 0.02 |
| HFI | Mule deer | **-0.51** | 0.08 | -0.21 |
| HFI | Red fox | **-0.47** | 0.05 | -0.21 |
| HFI | Red Squirrel | **-0.57** | 0.01 | -0.28 |
| HFI | Snowshoe Hare | **-0.06** | 0.59 | 0.26 |
| HFI | White-tailed deer | **-0.13** | 0.30 | 0.09 |
| Season winter | Black Bear | **-1.88** | -0.74 | -1.30 |
| Season winter | Canada Lynx | **-0.43** | 0.48 | 0.00 |
| Season winter | Cougar | **-0.75** | 0.56 | -0.12 |
| Season winter | Coyote | **-0.75** | 0.20 | -0.28 |
| Season winter | Elk wapiti | **-1.17** | 0.48 | -0.35 |
| Season winter | Gray Wolf | **-0.89** | 0.09 | -0.40 |
| Season winter | Grizzly bear | **-1.55** | -0.44 | -0.98 |
| Season winter | Ground squirrel | **-2.23** | 1.05 | -0.60 |
| Season winter | Marten | **-0.87** | 0.07 | -0.38 |
| Season winter | Moose | **-0.95** | 0.09 | -0.43 |
| Season winter | Mule deer | **-2.35** | -0.90 | -1.60 |
| Season winter | Red fox | **-0.70** | 0.30 | -0.19 |
| Season winter | Red Squirrel | **-0.40** | 0.71 | 0.14 |
| Season winter | Snowshoe Hare | **-0.41** | 0.77 | 0.16 |
| Season winter | White-tailed deer | **-1.59** | -0.49 | -1.03 |
| Elevation | Black Bear | **-0.73** | -0.24 | -0.48 |
| Elevation | Canada Lynx | **-0.60** | -0.08 | -0.34 |
| Elevation | Cougar | **-0.74** | -0.12 | -0.42 |
| Elevation | Coyote | **-0.94** | -0.45 | -0.69 |
| Elevation | Elk wapiti | **-0.73** | 0.05 | -0.35 |
| Elevation | Gray Wolf | **-0.46** | -0.03 | -0.24 |
| Elevation | Grizzly bear | **-0.20** | 0.27 | 0.04 |
| Elevation | Ground squirrel | **-0.20** | 1.25 | 0.49 |
| Elevation | Marten | **-0.29** | 0.22 | -0.04 |
| Elevation | Moose | **-0.30** | 0.16 | -0.07 |
| Elevation | Mule deer | **-0.14** | 0.40 | 0.12 |
| Elevation | Red fox | **-0.91** | -0.43 | -0.67 |
| Elevation | Red Squirrel | **-0.55** | 0.04 | -0.26 |
| Elevation | Snowshoe Hare | **-0.69** | -0.02 | -0.35 |
| Elevation | White-tailed deer | **-0.99** | -0.55 | -0.77 |
| Distance to water | Black Bear | **-0.12** | 0.28 | 0.08 |
| Distance to water | Canada Lynx | **-0.10** | 0.31 | 0.11 |
| Distance to water | Cougar | **-0.32** | 0.23 | -0.04 |
| Distance to water | Coyote | **0.02** | 0.39 | 0.20 |
| Distance to water | Elk wapiti | **-0.44** | 0.24 | -0.10 |
| Distance to water | Gray Wolf | **-0.12** | 0.25 | 0.06 |
| Distance to water | Grizzly bear | **-0.19** | 0.21 | 0.01 |
| Distance to water | Ground squirrel | **-0.81** | 0.48 | -0.15 |
| Distance to water | Marten | **-0.43** | 0.02 | -0.20 |
| Distance to water | Moose | **-0.40** | -0.03 | -0.21 |
| Distance to water | Mule deer | **-0.44** | 0.04 | -0.20 |
| Distance to water | Red fox | **-0.16** | 0.22 | 0.03 |
| Distance to water | Red Squirrel | **0.00** | 0.47 | 0.24 |
| Distance to water | Snowshoe Hare | **-0.01** | 0.50 | 0.24 |
| Distance to water | White-tailed deer | **-0.11** | 0.23 | 0.06 |
| Forest vegetation class | Black Bear | **-0.55** | 0.43 | -0.07 |
| Forest vegetation class | Canada Lynx | **-0.83** | 0.17 | -0.33 |
| Forest vegetation class | Cougar | **-1.27** | -0.12 | -0.69 |
| Forest vegetation class | Coyote | **-0.75** | 0.17 | -0.29 |
| Forest vegetation class | Elk wapiti | **-1.72** | -0.38 | -1.04 |
| Forest vegetation class | Gray Wolf | **-0.63** | 0.26 | -0.20 |
| Forest vegetation class | Grizzly bear | **-0.54** | 0.38 | -0.09 |
| Forest vegetation class | Ground squirrel | **-3.42** | -0.74 | -2.04 |
| Forest vegetation class | Marten | **-0.53** | 0.44 | -0.05 |
| Forest vegetation class | Moose | **-0.63** | 0.29 | -0.16 |
| Forest vegetation class | Mule deer | **-0.50** | 0.61 | 0.04 |
| Forest vegetation class | Red fox | **-0.87** | 0.05 | -0.40 |
| Forest vegetation class | Red Squirrel | **-0.84** | 0.19 | -0.31 |
| Forest vegetation class | Snowshoe Hare | **-0.68** | 0.41 | -0.13 |
| Forest vegetation class | White-tailed deer | **-0.22** | 0.80 | 0.30 |
| Grassland-Shrubland vegetation class | Black Bear | **-1.06** | 0.08 | -0.49 |
| Grassland-Shrubland vegetation class | Canada Lynx | **-1.49** | -0.28 | -0.87 |
| Grassland-Shrubland vegetation class | Cougar | **-1.57** | -0.26 | -0.91 |
| Grassland-Shrubland vegetation class | Coyote | **-1.10** | 0.01 | -0.53 |
| Grassland-Shrubland vegetation class | Elk wapiti | **-1.76** | -0.19 | -0.97 |
| Grassland-Shrubland vegetation class | Gray Wolf | **-1.35** | -0.24 | -0.78 |
| Grassland-Shrubland vegetation class | Grizzly bear | **-1.17** | -0.10 | -0.63 |
| Grassland-Shrubland vegetation class | Ground squirrel | **-2.69** | 0.24 | -1.20 |
| Grassland-Shrubland vegetation class | Marten | **-1.24** | -0.07 | -0.65 |
| Grassland-Shrubland vegetation class | Moose | **-1.05** | 0.02 | -0.51 |
| Grassland-Shrubland vegetation class | Mule deer | **-1.26** | 0.04 | -0.60 |
| Grassland-Shrubland vegetation class | Red fox | **-1.07** | 0.01 | -0.53 |
| Grassland-Shrubland vegetation class | Red Squirrel | **-1.28** | -0.05 | -0.66 |
| Grassland-Shrubland vegetation class | Snowshoe Hare | **-1.31** | -0.03 | -0.67 |
| Grassland-Shrubland vegetation class | White-tailed deer | **-0.87** | 0.36 | -0.25 |
| Other vegetation class | Black Bear | **-0.87** | 0.53 | -0.16 |
| Other vegetation class | Canada Lynx | **-0.98** | 0.36 | -0.30 |
| Other vegetation class | Cougar | **-0.86** | 0.67 | -0.09 |
| Other vegetation class | Coyote | **-0.74** | 0.57 | -0.08 |
| Other vegetation class | Elk wapiti | **-0.87** | 0.96 | 0.03 |
| Other vegetation class | Gray Wolf | **-0.59** | 0.63 | 0.02 |
| Other vegetation class | Grizzly bear | **-0.76** | 0.51 | -0.12 |
| Other vegetation class | Ground squirrel | **-1.74** | 1.88 | 0.03 |
| Other vegetation class | Marten | **-1.04** | 0.33 | -0.34 |
| Other vegetation class | Moose | **-0.63** | 0.66 | 0.01 |
| Other vegetation class | Mule deer | **-0.89** | 0.63 | -0.12 |
| Other vegetation class | Red fox | **-0.59** | 0.63 | 0.02 |
| Other vegetation class | Red Squirrel | **-0.95** | 0.47 | -0.24 |
| Other vegetation class | Snowshoe Hare | **-0.77** | 0.67 | -0.05 |
| Other vegetation class | White-tailed deer | **-0.89** | 0.58 | -0.17 |
| NDVI | Black Bear | **-0.23** | 0.41 | 0.10 |
| NDVI | Canada Lynx | **-0.26** | 0.28 | 0.01 |
| NDVI | Cougar | **-0.49** | 0.23 | -0.13 |
| NDVI | Coyote | **-0.39** | 0.17 | -0.11 |
| NDVI | Elk wapiti | **-0.32** | 0.61 | 0.13 |
| NDVI | Gray Wolf | **-0.54** | 0.02 | -0.26 |
| NDVI | Grizzly bear | **-0.51** | 0.08 | -0.21 |
| NDVI | Ground squirrel | **-0.66** | 1.17 | 0.18 |
| NDVI | Marten | **-0.43** | 0.15 | -0.14 |
| NDVI | Moose | **-0.03** | 0.54 | 0.25 |
| NDVI | Mule deer | **-0.34** | 0.40 | 0.03 |
| NDVI | Red fox | **-0.52** | 0.04 | -0.24 |
| NDVI | Red Squirrel | **-0.56** | 0.10 | -0.24 |
| NDVI | Snowshoe Hare | **-0.26** | 0.39 | 0.06 |
| NDVI | White-tailed deer | **-0.22** | 0.38 | 0.08 |
| Sampling Effort | Black Bear | **0.00** | 0.01 | 0.01 |
| Sampling Effort | Canada Lynx | **0.00** | 0.01 | 0.00 |
| Sampling Effort | Cougar | **0.00** | 0.01 | 0.01 |
| Sampling Effort | Coyote | **0.00** | 0.01 | 0.01 |
| Sampling Effort | Elk wapiti | **0.00** | 0.01 | 0.01 |
| Sampling Effort | Gray Wolf | **0.01** | 0.01 | 0.01 |
| Sampling Effort | Grizzly bear | **0.00** | 0.01 | 0.01 |
| Sampling Effort | Ground squirrel | **0.00** | 0.02 | 0.01 |
| Sampling Effort | Marten | **0.00** | 0.01 | 0.00 |
| Sampling Effort | Moose | **0.00** | 0.01 | 0.00 |
| Sampling Effort | Mule deer | **0.00** | 0.01 | 0.00 |
| Sampling Effort | Red fox | **0.00** | 0.01 | 0.01 |
| Sampling Effort | Red Squirrel | **0.00** | 0.01 | 0.01 |
| Sampling Effort | Snowshoe Hare | **0.00** | 0.01 | 0.01 |
| Sampling Effort | White-tailed deer | **0.00** | 0.01 | 0.01 |
| Strava: Non-motorized trail | Black Bear | **-0.08** | 0.00 | -0.04 |
| Strava: Non-motorized trail | Canada Lynx | **-0.08** | -0.01 | -0.04 |
| Strava: Non-motorized trail | Cougar | **-0.09** | 0.01 | -0.04 |
| Strava: Non-motorized trail | Coyote | **-0.02** | 0.04 | 0.01 |
| Strava: Non-motorized trail | Elk wapiti | **-0.09** | 0.02 | -0.04 |
| Strava: Non-motorized trail | Gray Wolf | **-0.06** | 0.02 | -0.02 |
| Strava: Non-motorized trail | Grizzly bear | **-0.09** | -0.01 | -0.05 |
| Strava: Non-motorized trail | Ground squirrel | **-0.21** | 0.03 | -0.09 |
| Strava: Non-motorized trail | Marten | **-0.07** | 0.01 | -0.03 |
| Strava: Non-motorized trail | Moose | **-0.04** | 0.02 | -0.01 |
| Strava: Non-motorized trail | Mule deer | **-0.12** | -0.01 | -0.06 |
| Strava: Non-motorized trail | Red fox | **-0.01** | 0.04 | 0.02 |
| Strava: Non-motorized trail | Red Squirrel | **-0.06** | 0.01 | -0.02 |
| Strava: Non-motorized trail | Snowshoe Hare | **-0.05** | 0.02 | -0.01 |
| Strava: Non-motorized trail | White-tailed deer | **-0.04** | 0.03 | -0.01 |
| Strava: Motorized | Black Bear | **-0.03** | 0.03 | 0.00 |
| Strava: Motorized | Canada Lynx | **-0.05** | 0.02 | -0.02 |
| Strava: Motorized | Cougar | **-0.06** | 0.03 | -0.01 |
| Strava: Motorized | Coyote | **-0.06** | 0.01 | -0.03 |
| Strava: Motorized | Elk wapiti | **-0.06** | 0.02 | -0.02 |
| Strava: Motorized | Gray Wolf | **-0.02** | 0.04 | 0.01 |
| Strava: Motorized | Grizzly bear | **-0.02** | 0.03 | 0.01 |
| Strava: Motorized | Ground squirrel | **-0.15** | 0.05 | -0.05 |
| Strava: Motorized | Marten | **-0.02** | 0.04 | 0.01 |
| Strava: Motorized | Moose | **-0.01** | 0.04 | 0.01 |
| Strava: Motorized | Mule deer | **-0.05** | 0.03 | -0.01 |
| Strava: Motorized | Red fox | **-0.03** | 0.02 | 0.00 |
| Strava: Motorized | Red Squirrel | **-0.05** | 0.02 | -0.01 |
| Strava: Motorized | Snowshoe Hare | **-0.03** | 0.04 | 0.01 |
| Strava: Motorized | White-tailed deer | **-0.02** | 0.03 | 0.01 |
| Strava: Forest % | Black Bear | **-0.01** | 0.01 | 0.00 |
| Strava: Forest % | Canada Lynx | **-0.02** | 0.00 | -0.01 |
| Strava: Forest % | Cougar | **-0.02** | 0.01 | 0.00 |
| Strava: Forest % | Coyote | **-0.01** | 0.01 | 0.00 |
| Strava: Forest % | Elk wapiti | **-0.02** | 0.01 | 0.00 |
| Strava: Forest % | Gray Wolf | **-0.01** | 0.01 | 0.00 |
| Strava: Forest % | Grizzly bear | **-0.01** | 0.01 | 0.00 |
| Strava: Forest % | Ground squirrel | **-0.05** | 0.02 | -0.01 |
| Strava: Forest % | Marten | **-0.01** | 0.01 | 0.00 |
| Strava: Forest % | Moose | **-0.01** | 0.01 | 0.00 |
| Strava: Forest % | Mule deer | **-0.01** | 0.01 | 0.00 |
| Strava: Forest % | Red fox | **-0.01** | 0.01 | 0.00 |
| Strava: Forest % | Red Squirrel | **-0.01** | 0.01 | 0.00 |
| Strava: Forest % | Snowshoe Hare | **-0.02** | 0.01 | 0.00 |
| Strava: Forest % | White-tailed deer | **0.00** | 0.02 | 0.01 |
| Strava:Mixed-use trail | Black Bear | **-0.01** | 0.01 | 0.00 |
| Strava:Mixed-use trail | Canada Lynx | **-0.01** | 0.01 | 0.00 |
| Strava:Mixed-use trail | Cougar | **-0.01** | 0.02 | 0.00 |
| Strava:Mixed-use trail | Coyote | **0.00** | 0.02 | 0.01 |
| Strava:Mixed-use trail | Elk wapiti | **-0.01** | 0.02 | 0.00 |
| Strava:Mixed-use trail | Gray Wolf | **-0.01** | 0.01 | 0.00 |
| Strava:Mixed-use trail | Grizzly bear | **-0.01** | 0.01 | 0.00 |
| Strava:Mixed-use trail | Ground squirrel | **-0.03** | 0.03 | 0.00 |
| Strava:Mixed-use trail | Marten | **-0.01** | 0.01 | 0.00 |
| Strava:Mixed-use trail | Moose | **0.00** | 0.02 | 0.01 |
| Strava:Mixed-use trail | Mule deer | **-0.01** | 0.02 | 0.01 |
| Strava:Mixed-use trail | Red fox | **0.00** | 0.02 | 0.01 |
| Strava:Mixed-use trail | Red Squirrel | **0.00** | 0.02 | 0.01 |
| Strava:Mixed-use trail | Snowshoe Hare | **0.00** | 0.02 | 0.01 |
| Strava:Mixed-use trail | White-tailed deer | **-0.01** | 0.01 | 0.00 |
| Strava: HFI | Black Bear | **-0.01** | 0.02 | 0.01 |
| Strava: HFI | Canada Lynx | **-0.02** | 0.02 | 0.00 |
| Strava: HFI | Cougar | **-0.02** | 0.02 | 0.00 |
| Strava: HFI | Coyote | **-0.02** | 0.01 | -0.01 |
| Strava: HFI | Elk wapiti | **-0.01** | 0.04 | 0.02 |
| Strava: HFI | Gray Wolf | **-0.01** | 0.02 | 0.00 |
| Strava: HFI | Grizzly bear | **0.00** | 0.03 | 0.01 |
| Strava: HFI | Ground squirrel | **-0.03** | 0.06 | 0.01 |
| Strava: HFI | Marten | **-0.01** | 0.02 | 0.00 |
| Strava: HFI | Moose | **0.00** | 0.03 | 0.02 |
| Strava: HFI | Mule deer | **-0.02** | 0.02 | 0.00 |
| Strava: HFI | Red fox | **-0.02** | 0.01 | 0.00 |
| Strava: HFI | Red Squirrel | **-0.02** | 0.02 | 0.00 |
| Strava: HFI | Snowshoe Hare | **-0.03** | 0.00 | -0.01 |
| Strava: HFI | White-tailed deer | **-0.01** | 0.02 | 0.00 |
| Strava: Season winter | Black Bear | **-0.04** | 0.00 | -0.02 |
| Strava: Season winter | Canada Lynx | **-0.01** | 0.01 | 0.00 |
| Strava: Season winter | Cougar | **-0.05** | 0.02 | -0.01 |
| Strava: Season winter | Coyote | **-0.02** | 0.02 | 0.00 |
| Strava: Season winter | Elk wapiti | **-0.04** | 0.03 | 0.00 |
| Strava: Season winter | Gray Wolf | **-0.03** | 0.02 | 0.00 |
| Strava: Season winter | Grizzly bear | **-0.04** | 0.01 | -0.01 |
| Strava: Season winter | Ground squirrel | **-0.11** | 0.04 | -0.03 |
| Strava: Season winter | Marten | **-0.02** | 0.01 | 0.00 |
| Strava: Season winter | Moose | **-0.02** | 0.02 | 0.00 |
| Strava: Season winter | Mule deer | **-0.04** | 0.02 | -0.01 |
| Strava: Season winter | Red fox | **-0.02** | 0.02 | 0.00 |
| Strava: Season winter | Red Squirrel | **-0.03** | 0.01 | -0.01 |
| Strava: Season winter | Snowshoe Hare | **-0.01** | 0.03 | 0.01 |
| Strava: Season winter | White-tailed deer | **-0.01** | 0.03 | 0.01 |
| Distance to trail: Motorized trails | Black Bear | **-0.85** | 4.07 | 1.60 |
| Distance to trail: Motorized trails | Canada Lynx | **-4.59** | 0.40 | -2.04 |
| Distance to trail: Motorized trails | Cougar | **-3.87** | 1.28 | -1.29 |
| Distance to trail: Motorized trails | Coyote | **-2.16** | 2.51 | 0.16 |
| Distance to trail: Motorized trails | Elk wapiti | **-1.66** | 4.48 | 1.37 |
| Distance to trail: Motorized trails | Gray Wolf | **-3.76** | 0.90 | -1.47 |
| Distance to trail: Motorized trails | Grizzly bear | **-2.95** | 1.73 | -0.66 |
| Distance to trail: Motorized trails | Ground squirrel | **-2.44** | 7.72 | 2.51 |
| Distance to trail: Motorized trails | Marten | **-3.48** | 1.55 | -0.96 |
| Distance to trail: Motorized trails | Moose | **-2.43** | 2.30 | -0.09 |
| Distance to trail: Motorized trails | Mule deer | **-0.57** | 4.75 | 2.10 |
| Distance to trail: Motorized trails | Red fox | **-3.51** | 1.26 | -1.16 |
| Distance to trail: Motorized trails | Red Squirrel | **-3.99** | 1.07 | -1.46 |
| Distance to trail: Motorized trails | Snowshoe Hare | **-4.49** | 0.59 | -1.95 |
| Distance to trail: Motorized trails | White-tailed deer | **-1.58** | 3.49 | 0.99 |
| Distance to trail: Motorized trails | Black Bear | **-0.80** | 3.39 | 1.30 |
| Distance to trail: Motorized trails | Canada Lynx | **-2.15** | 2.22 | 0.01 |
| Distance to trail: Motorized trails | Cougar | **-2.01** | 2.89 | 0.39 |
| Distance to trail: Motorized trails | Coyote | **-2.18** | 1.91 | -0.13 |
| Distance to trail: Motorized trails | Elk wapiti | **-3.56** | 1.90 | -0.81 |
| Distance to trail: Motorized trails | Gray Wolf | **-1.60** | 2.53 | 0.47 |
| Distance to trail: Motorized trails | Grizzly bear | **-1.67** | 2.43 | 0.39 |
| Distance to trail: Motorized trails | Ground squirrel | **-5.38** | 4.01 | -0.64 |
| Distance to trail: Motorized trails | Marten | **-0.56** | 3.66 | 1.58 |
| Distance to trail: Motorized trails | Moose | **-0.77** | 3.51 | 1.35 |
| Distance to trail: Motorized trails | Mule deer | **-0.30** | 4.45 | 2.06 |
| Distance to trail: Motorized trails | Red fox | **-1.28** | 2.87 | 0.80 |
| Distance to trail: Motorized trails | Red Squirrel | **-2.03** | 2.36 | 0.17 |
| Distance to trail: Motorized trails | Snowshoe Hare | **-2.73** | 1.87 | -0.46 |
| Distance to trail: Motorized trails | White-tailed deer | **-1.74** | 2.81 | 0.57 |
| Distance: Forest % | Black Bear | **-0.47** | 0.31 | -0.07 |
| Distance: Forest % | Canada Lynx | **-0.16** | 0.63 | 0.24 |
| Distance: Forest % | Cougar | **-0.34** | 0.71 | 0.16 |
| Distance: Forest % | Coyote | **-0.37** | 0.37 | 0.00 |
| Distance: Forest % | Elk wapiti | **-0.42** | 0.96 | 0.25 |
| Distance: Forest % | Gray Wolf | **-0.23** | 0.50 | 0.14 |
| Distance: Forest % | Grizzly bear | **-0.30** | 0.53 | 0.12 |
| Distance: Forest % | Ground squirrel | **-1.03** | 1.83 | 0.37 |
| Distance: Forest % | Marten | **-0.08** | 0.82 | 0.35 |
| Distance: Forest % | Moose | **-0.21** | 0.53 | 0.17 |
| Distance: Forest % | Mule deer | **0.02** | 1.12 | 0.55 |
| Distance: Forest % | Red fox | **-0.04** | 0.72 | 0.34 |
| Distance: Forest % | Red Squirrel | **-0.28** | 0.58 | 0.16 |
| Distance: Forest % | Snowshoe Hare | **-0.67** | 0.32 | -0.17 |
| Distance: Forest % | White-tailed deer | **-0.37** | 0.27 | -0.05 |
| Distance to trail: Management | Black Bear | **-0.43** | 0.05 | -0.19 |
| Distance to trail: Management | Canada Lynx | **-0.20** | 0.28 | 0.03 |
| Distance to trail: Management | Cougar | **-0.36** | 0.26 | -0.05 |
| Distance to trail: Management | Coyote | **-0.33** | 0.16 | -0.09 |
| Distance to trail: Management | Elk wapiti | **-0.49** | 0.22 | -0.14 |
| Distance to trail: Management | Gray Wolf | **-0.30** | 0.19 | -0.06 |
| Distance to trail: Management | Grizzly bear | **-0.32** | 0.13 | -0.10 |
| Distance to trail: Management | Ground squirrel | **-0.78** | 0.50 | -0.13 |
| Distance to trail: Management | Marten | **-0.50** | 0.01 | -0.23 |
| Distance to trail: Management | Moose | **-0.40** | 0.06 | -0.18 |
| Distance to trail: Management | Mule deer | **-0.64** | -0.05 | -0.35 |
| Distance to trail: Management | Red fox | **-0.38** | 0.12 | -0.13 |
| Distance to trail: Management | Red Squirrel | **-0.23** | 0.32 | 0.04 |
| Distance to trail: Management | Snowshoe Hare | **-0.28** | 0.30 | 0.01 |
| Distance to trail: Management | White-tailed deer | **-0.47** | 0.06 | -0.21 |
| Distance to trail: HFI | Black Bear | **-0.08** | 0.36 | 0.14 |
| Distance to trail: HFI | Canada Lynx | **-0.23** | 0.20 | -0.02 |
| Distance to trail: HFI | Cougar | **-0.20** | 0.40 | 0.11 |
| Distance to trail: HFI | Coyote | **0.01** | 0.41 | 0.21 |
| Distance to trail: HFI | Elk wapiti | **-0.25** | 0.55 | 0.15 |
| Distance to trail: HFI | Gray Wolf | **-0.06** | 0.35 | 0.15 |
| Distance to trail: HFI | Grizzly bear | **-0.30** | 0.24 | -0.02 |
| Distance to trail: HFI | Ground squirrel | **-0.41** | 1.19 | 0.34 |
| Distance to trail: HFI | Marten | **-0.44** | 0.10 | -0.17 |
| Distance to trail: HFI | Moose | **-0.49** | 0.04 | -0.22 |
| Distance to trail: HFI | Mule deer | **-0.37** | 0.31 | -0.02 |
| Distance to trail: HFI | Red fox | **-0.17** | 0.27 | 0.05 |
| Distance to trail: HFI | Red Squirrel | **-0.38** | 0.15 | -0.11 |
| Distance to trail: HFI | Snowshoe Hare | **-0.39** | 0.17 | -0.11 |
| Distance to trail: HFI | White-tailed deer | **-0.14** | 0.25 | 0.05 |
| Distance: Season winter | Black Bear | **-1.46** | -0.51 | -0.97 |
| Distance: Season winter | Canada Lynx | **-0.48** | -0.04 | -0.25 |
| Distance: Season winter | Cougar | **-0.59** | 0.41 | -0.07 |
| Distance: Season winter | Coyote | **-0.30** | 0.24 | -0.03 |
| Distance: Season winter | Elk wapiti | **-1.49** | 0.00 | -0.72 |
| Distance: Season winter | Gray Wolf | **-0.76** | -0.04 | -0.39 |
| Distance: Season winter | Grizzly bear | **-1.15** | -0.15 | -0.63 |
| Distance: Season winter | Ground squirrel | **-2.94** | 0.23 | -1.24 |
| Distance: Season winter | Marten | **-0.22** | 0.28 | 0.03 |
| Distance: Season winter | Moose | **-1.29** | -0.40 | -0.83 |
| Distance: Season winter | Mule deer | **-2.00** | -0.63 | -1.27 |
| Distance: Season winter | Red fox | **-0.38** | 0.21 | -0.09 |
| Distance: Season winter | Red Squirrel | **-0.15** | 0.41 | 0.12 |
| Distance: Season winter | Snowshoe Hare | **-0.31** | 0.31 | 0.00 |
| Distance: Season winter | White-tailed deer | **-0.61** | -0.03 | -0.32 |
| Density trail: Non-motorized trail | Black Bear | **0.03** | 1.10 | 0.56 |
| Density trail: Non-motorized trail | Canada Lynx | **-0.58** | 0.97 | 0.22 |
| Density trail: Non-motorized trail | Cougar | **-0.69** | 1.03 | 0.17 |
| Density trail: Non-motorized trail | Coyote | **0.21** | 1.35 | 0.77 |
| Density trail: Non-motorized trail | Elk wapiti | **-0.83** | 1.23 | 0.22 |
| Density trail: Non-motorized trail | Gray Wolf | **0.19** | 1.35 | 0.75 |
| Density trail: Non-motorized trail | Grizzly bear | **-0.40** | 0.87 | 0.26 |
| Density trail: Non-motorized trail | Ground squirrel | **-2.11** | 1.88 | -0.09 |
| Density trail: Non-motorized trail | Marten | **-0.54** | 0.73 | 0.10 |
| Density trail: Non-motorized trail | Moose | **-0.06** | 0.96 | 0.45 |
| Density trail: Non-motorized trail | Mule deer | **-0.43** | 1.06 | 0.32 |
| Density trail: Non-motorized trail | Red fox | **-0.06** | 1.04 | 0.48 |
| Density trail: Non-motorized trail | Red Squirrel | **0.05** | 1.27 | 0.65 |
| Density trail: Non-motorized trail | Snowshoe Hare | **-0.30** | 1.19 | 0.47 |
| Density trail: Non-motorized trail | White-tailed deer | **0.16** | 1.27 | 0.72 |
| Density: Motorized trails | Black Bear | **-0.12** | 1.03 | 0.45 |
| Density: Motorized trails | Canada Lynx | **0.08** | 1.40 | 0.71 |
| Density: Motorized trails | Cougar | **0.15** | 1.67 | 0.90 |
| Density: Motorized trails | Coyote | **0.29** | 1.46 | 0.86 |
| Density: Motorized trails | Elk wapiti | **-0.53** | 1.29 | 0.38 |
| Density: Motorized trails | Gray Wolf | **0.02** | 1.24 | 0.62 |
| Density: Motorized trails | Grizzly bear | **0.06** | 1.23 | 0.64 |
| Density: Motorized trails | Ground squirrel | **-1.42** | 2.07 | 0.36 |
| Density: Motorized trails | Marten | **0.07** | 1.23 | 0.64 |
| Density: Motorized trails | Moose | **-0.32** | 0.83 | 0.25 |
| Density: Motorized trails | Mule deer | **-0.49** | 0.86 | 0.19 |
| Density: Motorized trails | Red fox | **-0.03** | 1.13 | 0.53 |
| Density: Motorized trails | Red Squirrel | **0.14** | 1.42 | 0.76 |
| Density: Motorized trails | Snowshoe Hare | **0.09** | 1.46 | 0.75 |
| Density: Motorized trails | White-tailed deer | **-0.04** | 1.22 | 0.59 |
| Distance to trail: Forest % | Black Bear | **-0.49** | 0.36 | -0.06 |
| Distance to trail: Forest % | Canada Lynx | **-0.25** | 0.52 | 0.14 |
| Distance to trail: Forest % | Cougar | **-0.02** | 1.11 | 0.52 |
| Distance to trail: Forest % | Coyote | **-0.15** | 0.67 | 0.26 |
| Distance to trail: Forest % | Elk wapiti | **-0.25** | 1.05 | 0.37 |
| Distance to trail: Forest % | Gray Wolf | **0.11** | 1.02 | 0.56 |
| Distance to trail: Forest % | Grizzly bear | **-0.01** | 0.80 | 0.40 |
| Distance to trail: Forest % | Ground squirrel | **-0.85** | 1.65 | 0.39 |
| Distance to trail: Forest % | Marten | **-0.15** | 0.78 | 0.31 |
| Distance to trail: Forest % | Moose | **0.04** | 0.81 | 0.43 |
| Distance to trail: Forest % | Mule deer | **-0.30** | 0.77 | 0.23 |
| Distance to trail: Forest % | Red fox | **-0.18** | 0.70 | 0.26 |
| Distance to trail: Forest % | Red Squirrel | **-0.30** | 0.71 | 0.20 |
| Distance to trail: Forest % | Snowshoe Hare | **-0.34** | 0.68 | 0.17 |
| Distance to trail: Forest % | White-tailed deer | **-0.41** | 0.37 | -0.02 |
| Density trail: Management | Black Bear | **-0.58** | -0.07 | -0.33 |
| Density trail: Management | Canada Lynx | **-0.65** | -0.14 | -0.39 |
| Density trail: Management | Cougar | **-0.64** | -0.08 | -0.36 |
| Density trail: Management | Coyote | **-0.66** | -0.17 | -0.42 |
| Density trail: Management | Elk wapiti | **-0.69** | -0.05 | -0.37 |
| Density trail: Management | Gray Wolf | **-0.59** | -0.10 | -0.34 |
| Density trail: Management | Grizzly bear | **-0.57** | -0.08 | -0.33 |
| Density trail: Management | Ground squirrel | **-0.83** | 0.30 | -0.27 |
| Density trail: Management | Marten | **-0.54** | -0.04 | -0.29 |
| Density trail: Management | Moose | **-0.59** | -0.10 | -0.35 |
| Density trail: Management | Mule deer | **-0.56** | 0.00 | -0.28 |
| Density trail: Management | Red fox | **-0.61** | -0.12 | -0.37 |
| Density trail: Management | Red Squirrel | **-0.62** | -0.10 | -0.36 |
| Density trail: Management | Snowshoe Hare | **-0.70** | -0.18 | -0.44 |
| Density trail: Management | White-tailed deer | **-0.70** | -0.14 | -0.42 |
| Density trail: HFI | Black Bear | **-0.52** | 0.22 | -0.15 |
| Density trail: HFI | Canada Lynx | **0.10** | 0.79 | 0.44 |
| Density trail: HFI | Cougar | **-0.01** | 0.93 | 0.44 |
| Density trail: HFI | Coyote | **-0.28** | 0.41 | 0.06 |
| Density trail: HFI | Elk wapiti | **-0.13** | 0.94 | 0.39 |
| Density trail: HFI | Gray Wolf | **-0.24** | 0.47 | 0.13 |
| Density trail: HFI | Grizzly bear | **-0.20** | 0.51 | 0.15 |
| Density trail: HFI | Ground squirrel | **-0.53** | 1.54 | 0.50 |
| Density trail: HFI | Marten | **-0.09** | 0.65 | 0.27 |
| Density trail: HFI | Moose | **-0.06** | 0.62 | 0.28 |
| Density trail: HFI | Mule deer | **-0.79** | 0.12 | -0.33 |
| Density trail: HFI | Red fox | **-0.45** | 0.29 | -0.08 |
| Density trail: HFI | Red Squirrel | **0.13** | 0.94 | 0.53 |
| Density trail: HFI | Snowshoe Hare | **-0.01** | 0.82 | 0.40 |
| Density trail: HFI | White-tailed deer | **-0.54** | 0.20 | -0.17 |
| Density trail: Season winter | Black Bear | **-0.23** | 0.68 | 0.23 |
| Density trail: Season winter | Canada Lynx | **-0.19** | 0.40 | 0.09 |
| Density trail: Season winter | Cougar | **-0.51** | 0.64 | 0.07 |
| Density trail: Season winter | Coyote | **-0.25** | 0.58 | 0.15 |
| Density trail: Season winter | Elk wapiti | **-0.73** | 0.79 | 0.03 |
| Density trail: Season winter | Gray Wolf | **-0.62** | 0.32 | -0.15 |
| Density trail: Season winter | Grizzly bear | **-0.39** | 0.52 | 0.07 |
| Density trail: Season winter | Ground squirrel | **-1.56** | 1.39 | -0.06 |
| Density trail: Season winter | Marten | **-0.57** | 0.16 | -0.21 |
| Density trail: Season winter | Moose | **-0.26** | 0.64 | 0.19 |
| Density trail: Season winter | Mule deer | **-0.12** | 1.06 | 0.47 |
| Density trail: Season winter | Red fox | **-0.40** | 0.46 | 0.03 |
| Density trail: Season winter | Red Squirrel | **-0.57** | 0.29 | -0.13 |
| Density trail: Season winter | Snowshoe Hare | **-0.61** | 0.30 | -0.15 |
| Density trail: Season winter | White-tailed deer | **-0.37** | 0.54 | 0.09 |
