# Supplementary material for: Mammal responses to human recreation depend on landscape context
Source: PLoS One. 2024 Jul 18;19(7):e0300870. doi: 10.1371/journal.pone.0300870 (PMC11257333; doi:10.1371/journal.pone.0300870)
Supplement: S1 File — (DOCX) [file pone.0300870.s003.docx]

**S1 File: Data Sources in relation to table 1 in the main text**

**Distance from trail and density of trail:** <https://geodiscover.alberta.ca/geoportal/rest/metadata/item/9ceba2a857a64719819f511ceeb6d234/html> and osmdata R package

**AllTrails**: <https://www.alltrails.com/>

**Strava:** <https://www.strava.com> Strava’s REST API (v3), using the stravalib library (v0.10.4) programmed in Python (v3.10.1)

**Trail designation :** <https://geodiscover.alberta.ca/geoportal/rest/metadata/item/9ceba2a857a64719819f511ceeb6d234/html>

**% Forest cover:** https://open.canada.ca/data/en/dataset/11990a35-912e-4002-b197-d57dd88836d7

**% Human Footprint:** <https://www.abmi.ca/home/data-analytics/da-top/da-product-overview/Human-Footprint-Products/HF-inventory.html>

**Type of management:** <https://open.alberta.ca/opendata>

**Elevation**: Elevatr R package (Hollister et al. 2021)

**Distance to water:** <https://www.altalis.com/>

**Land cover:** https://open.canada.ca/data/en/dataset/11990a35-912e-4002-b197-d57dd88836d7

**NDVI:** <https://appeears.earthdatacloud.nasa.gov/>
