# Supplementary material for: Mammal responses to human recreation depend on landscape context
Source: PLoS One. 2024 Jul 18;19(7):e0300870. doi: 10.1371/journal.pone.0300870 (PMC11257333; doi:10.1371/journal.pone.0300870)
Supplement: S1 Fig — %HF: % Human footprint, Dist: Distance, Dens = Density. See Table 1 for more details about variables. (DOCX) [file pone.0300870.s004.docx]

**S1 Fig.** Pearson correlation coefficients between quantitative variables used in the HMSC model to assess the influence of recreation and other factors on mammal habitat use. %HF: % Human footprint, Dist: Distance, Dens=Density. See Table 1 for more details about variables
