# Supplementary material for: Mammal responses to human recreation depend on landscape context
Source: PLoS One. 2024 Jul 18;19(7):e0300870. doi: 10.1371/journal.pone.0300870 (PMC11257333; doi:10.1371/journal.pone.0300870)

**S2 Fig.** Factor Analysis of Mixed Data (FAMD) assessing the similarity between all our variables See Table 1 for more details about variables. The left panel represents the correlation circle that shows the relationship between quantitative variables, the quality of the representation of variables, and the correlation between variables and the dimensions. The right panel represents the correlation between variables, quantitative and qualitative variables (in red), and the principal dimensions.


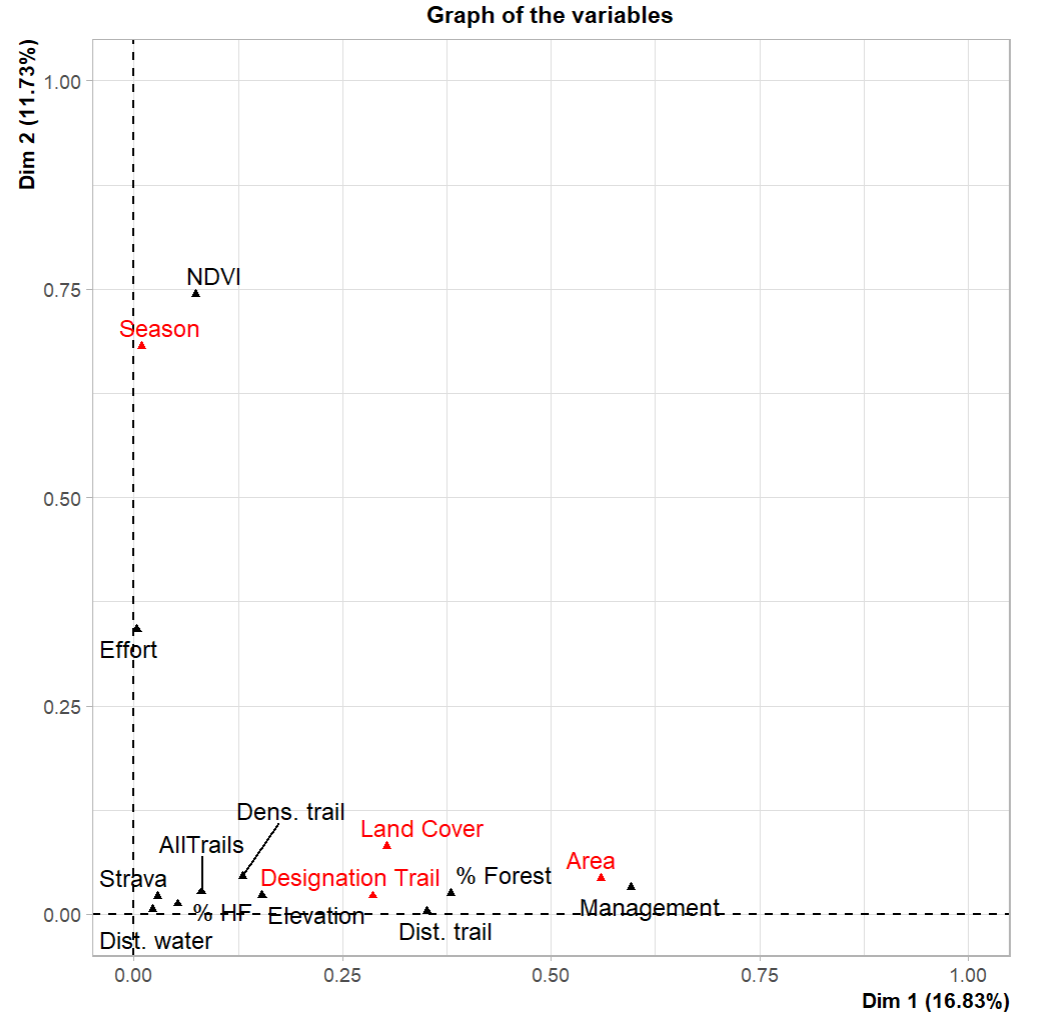

Supplement: S2 Fig — The left panel represents the correlation circle that shows the relationship between quantitative variables, the quality of the representation of variables, and the correlation between variables and the dimensions. The right panel represents the correlation between variables, quantitative and qualitative variables (in red), and the principal dimensions. (DOCX) [file pone.0300870.s005.docx]
