# Supplementary material for: Mammal responses to human recreation depend on landscape context
Source: PLoS One. 2024 Jul 18;19(7):e0300870. doi: 10.1371/journal.pone.0300870 (PMC11257333; doi:10.1371/journal.pone.0300870)
Supplement: S3 Fig — Responses with at least 95% posterior probability are shown in red with a “+” or in blue with a “-”depending if the relationship is positive (above 0) or negative (below 0). Predators are in orange, ungulates in green and small mammals in pink. Vertical lines separate the different recreation measures. (DOCX) [file pone.0300870.s006.docx]

**S3 Fig.** *Species responses to recreation, influencing factors, landscape variables and interaction between recreation and influencing factors with factors. Responses with at least 95% posterior probability are shown in red with a “+” or in blue with a “-“ depending if the relationship is positive (above 0) or negative (below 0). Predators are in orange, ungulates in green and small mammals in pink. Vertical lines separate the different recreation measures.*
