# Supplementary material for: Mammal responses to human recreation depend on landscape context
Source: PLoS One. 2024 Jul 18;19(7):e0300870. doi: 10.1371/journal.pone.0300870 (PMC11257333; doi:10.1371/journal.pone.0300870)
Supplement: Attachment — Submitted filename: Review for PLOS ONE Mammal responses to human recreation depend to landscape context.pdf [file pone.0300870.s007.pdf]

Review for PLOS ONE Mammal responses to human recreation depend to landscape context

I really enjoyed reviewing this manuscript, that I found very interesting, very well written and using an original modelling approach. Results are relevant and will certainly contribute to the growing body of evidence regarding animal responses to outdoor activities and recreation, a potential impact of growing presence and intensity across the globe, with increasing conservation concern. However there is one main point that the authors did not consider that I think might hamper the results presented in the manuscript and several minor comments.

My main doubt regards the methodology used to extract the variable describing recreation intensity, namely the Strava variable. Corradini et al. 2021, that you cite, used a different approach to extract an index based on Strava data: they extracted and rasterised the Strava map, rather than using the number of reviews by the users. I am not sure that these two methods are equivalent, does the number of reviews by users really reflect the intensity shown by Strava in their maps? Furthermore, I have an even more serious concern regarding this point: how can you be sure that your index based on Strava data realistically reflects the intensity of recreation? This data certainly needs a validation! Corradini et al. 2021 validated their index using camera-trapping based on trails and found a positive correlation. But this positive correlation needs to be proven locally in every study area one may choose, since it is not guaranteed that Strava data will thoroughly reflect real on-the-ground frequentation of trails. To sum up: 1- I am not sure your method is equivalent to Corradini et al. 2021 one and equally representative; 2 – In any case this index should be validated against another method to make sure it gives a reasonably faithful picture of recreation in your area.

Other comments:

Lines 82-87: The whole introduction is interesting, well written and well documented. However here I think you lack at least to mention that also hunting is a major factor that can influence avoidance behaviours in mammals. Much research shows that fear of humans can be influenced by the presence and intensity of hunting in a specific area.

Lines 104 – 112: Here I would strongly suggest to discuss the potential long-term effects that recreation might have on wildlife species, communities and ecosystem functions. Even when we observe clear avoidance behaviors in mammals towards human recreation we do not know if this avoidance leads to decreased fitness and hence diminishing population trends. One study that evaluated this aspect is *Salvatori, M., Oberosler, V., Rinaldi, M., Franceschini, A., Truschi, S., Pedrini, P., & Rovero, F. (2023). Crowded mountains: Long-term effects of human outdoor recreation on a community of wild mammals monitored with systematic camera trapping. Ambio, 52(6), 1085-1097*. *https://link.springer.com/article/10.1007/s13280-022-01825-w*, whose results are relevant also for the interpretation of you results in this manuscript.

Additionally, I think that here or in the discussion another point that should be explored is whether recreation can alter herbivory and predation patterns, resulting in direct consequences on other ecosystem components: a relevant subject also for protected area managers. One relevant work here is *Di Nicola, W., Mols, B., & Smit, C. (2023). Human recreation shapes the local scale impact of ungulates on the carbon pools of a temperate coniferous forest. Global Ecology and Conservation, 46, e02574. https://www.sciencedirect.com/science/article/pii/S2351989423002093*

Lines 183: Here you say that minimum distance from a trail was 50 meters but in Table 1 the minimum distances you report for the 2 areas are well below 50 meters.

Line 188: I do not understand why you lured your camera trapping sites. The lure effect will have faded a lot before your sampling was ended, since you kept your cameras for more than one year. Luring the site would

in this case bias sampling with a higher detection probability at the start of sampling that would have decreased as time passed by. Luring could also introduce biases towards certain species more attracted to the scent, and since you are using community modelling I do not think this is a wise choice.

192: So you controlled cameras only once per year? Didn't this result in many battery failure and SD card filling?

242: I wonder if one could merge the two types of info, distance from trail and recreation intensity on that trail into a single index weighting both factors together, since animals could remain more distant specifically to those trails more intensely visited by humans. If I am not wrong you did not try the interaction Distance:Strava.

265: This sentence is not clear: what do you mean by entire camera trap sampling area? The area sampled by one single camera trap or the whole study area?

Line 274: See my main concern regarding validation of the Strava data.

Line 370: Camera days can also be conveniently shaped as an offset.

Line 414: you accidentally put 2 'with' at the end of this line.

Line 437: I would put '(see Figure 2)' to avoid confusion.

Line 488: This last sentence is quite cryptic: why did you used it in combination with non-motorised trails then if it does not reflect vehicle passages at all?

Line 523: Again, I feel you here overlook the possible effect of hunting in determining avoidance behaviours.

Line 532-534: These two possible explanations seem completely in contrast one another.

Line 539: I wonder whether this lack of effect derives from the weakness of the Strava variable you extracted in representing real recreation. Alternatively, it could be that your study areas have relatively low recreation intensity rates, compared to more human dominated landscapes like those of western Europe. Comparing and discussing the responses to recreation between North America and western Europe could also be very interesting.

Line 546: What do you mean with this? What does this difference in home range size entail?

Lines 555 – 557: I did not understand what you mean here with leveraging, please clarify.

Line 622: 'tpressure' typo.

Line 658: 'ewithin' typo.

Line 664: Here you wisely mention the potential long-term effect on animal population demography potentially driven by avoidance of human recreation: see the paper I cited above that tackled this topic.
